# Why Is There an Increased Risk for Sudden Cardiac Death in Patients With Early Repolarization Syndrome?

**DOI:** 10.7759/cureus.26820

**Published:** 2022-07-13

**Authors:** Shreyas Yakkali, Sneha Teresa Selvin, Sonu Thomas, Viktoriya Bikeyeva, Ahmed Abdullah, Aleksandra Radivojevic, Anas A Abu Jad, Anvesh Ravanavena, Chetna Ravindra, Emmanuelar O Igweonu-Nwakile, Safina Ali, Salomi Paul, Pousette Hamid

**Affiliations:** 1 Internal Medicine, California Institute of Behavioral Neurosciences & Psychology, Fairfield, USA; 2 Behavioral Neurosciences and Psychology, California Institute of Behavioral Neurosciences & Psychology, Fairfield, USA; 3 General Surgery, California Institute of Behavioral Neurosciences & Psychology, Fairfield, USA; 4 Neurology, California Institute of Behavioral Neurosciences & Psychology, Fairfield, USA

**Keywords:** idiopathic ventricular fibrillation, early repolarization pattern, electrocardiogram (ecg/ekg), j wave syndromes, early repolarization syndrome, cardiac sudden death

## Abstract

The last two decades have changed the viewpoint on early repolarization syndrome (ERS). The prevalence of the early repolarization pattern is variable and ranges between 3-24% depending upon age, gender, and criteria used for J-point upliftment from baseline (0.05mV vs. 1 mV). While this pattern was previously linked with a benign result, multiple recent investigations have found a link between early repolarization and Sudden Cardiac Death (SCD) by causing life-threatening arrhythmias like Ventricular tachycardia/Ventricular fibrillation, a condition known as early repolarization syndrome. The syndrome falls under a broader bracket of J wave syndromes, which can be caused by early repolarization or depolarization abnormalities. The characteristics of early repolarization that are considered high risk for Sudden Cardiac Death include the amplitude of J-point upliftment from baseline ( > 0.2 mV), Inferior-lateral location of Early Repolarization pattern, and horizontal and downsloping ST-segment. Patients with symptomatic early repolarisation patterns on ECG are more likely to have repeated cardiac episodes. Implantable Cardioverter-Defibrillator (ICD) implantation and isoproterenol are the recommended treatments in symptomatic patients. On the other hand, asymptomatic patients with early repolarization patterns are prevalent and have a better outcome. Risk categorization is still obscure in asymptomatic early repolarization patterns. This traditional review outlines the known knowledge of pathophysiology behind the increased risk of sudden cardiac death, risk stratification of patients with ERS, and the treatment guidelines for patients with ERS. Further prospective studies are recommended to elucidate the exact mechanism for ventricular arrhythmogenesis in ERS patients and to risk stratifying asymptomatic patients with ERS.

## Introduction and background

Sudden cardiac death (SCD) is an untoward natural death from a cause of cardiac origin in a patient without any preceding disease that would result in a quick fatality within a brief period, often one hour from the beginning of symptoms [1]. A structurally damaged heart is linked to the majority of SCDs. The fatal arrhythmias leading to SCD are usually due to Ventricular Tachycardia (VT) that progresses into Ventricular fibrillation(VF) and then to asystole. This happens particularly in patients with advanced heart disease. In patients without structural heart disease, Polymorphic ventricular tachycardia is caused by various genetic and ion-channelopathies that contribute to life-threatening arrhythmias [1]. Primary electrophysiological disorders cause 10% of SCDs with known or unknown ion-channelopathies. Early repolarization is an example of an unknown ion-channel abnormality [2-4].

Early repolarization (ER)/ Early Repolarization Pattern (ERP) is a form of repolarization that happens due to an elevation of the junction between the QRS complex's termination and the start of the ST segment in two contiguous ECG leads. It is an electrocardiographic anomaly, also known as "J-wave" or "J- point elevation" [5]. The first to use the term Early Repolarization to explain ST-segment abnormalities and adjoining inversion of T wave, with early repolarization as the reason underlying it, was in 1951 [6]. ER is defined as J-point advancement above the baseline exhibited as either QRS complex slurring (during the transition from QRS-ST) or notching (a positive waveform formed on the end of the S wave), ST-segment elevation with concavity while upwards, and strong T-waves in the least of all two contiguous ECG leads [2]. Until the year 2000, the so-called "early repolarization pattern" was universally and unmistakably considered "normal," "normal variation," or "benign anomaly" [7]. However, recent studies have pointed out a link between Early Repolarization and an increased risk of sudden cardiac arrhythmia-related death [3,8-15]. This traditional review aims to help encapsulate the current state of knowledge on the early repolarization pattern and the pathophysiology behind its association with Sudden Cardiac Death.

## Review

Historical Perspective

Shipley and Hallaran were the first to describe an early ST-segment elevation in ECGs, obtained from 200 young, healthy people in 1936. The researchers noticed this in 25 percent of male participants and 16 percent of female participants in the study [16]. Similar results were discovered two years later, in 1938, on the surface ECG of a person who died of hypothermia [17]. Wasserburger et al. defined ER in 1961 as a one to four-millimeter elevation in the ST segment at the end of the QRS complex along with a prominent slur or notch in the V3-V6 precordial leads on the downsloping of the R wave [18]. The proposal that Early Repolarisation could be malignant in some cases was put forward in 1999. The researchers observed that in wedge preparations supplied by arterial blood, the Early Repolarization pattern could easily convert to one that causes polymorphic VT [19]. Kalla et al. and Takagi et al. provided the evidence supporting the above hypothesis in 2000; they reported that subjects with prominent ST-segment and J-wave upliftment from the baseline in inferior leads without SHD (structural heart disease) showed VF. They also thought that idiopathic Ventricular Fibrillation with an Early Repolarisation pattern in inferior ECG leads could be another form of the Brugada syndrome [20,21].

Several clinical reports during the last two decades (Japan, being the origin for the majority of those) have reported patients who had a sudden cardiac death and irregular J waves; however, in patients who were diagnosed with idiopathic Ventricular Fibrillation, the only abnormal result recorded was Early Repolarization [20-24]. Meanwhile, a few experimental studies have shown that ER has the potential to cause arrhythmia. The findings in this study also suggested that ER could potentially be harmful [7,25]. In 2007-2008, Hassaguerre et al. documented a significant ER incidence in individuals with idiopathic VF, which was more clinically sound and a turning point in our understanding of ER. The presence of ER was found in 64/206 (31 percent) of idiopathic Ventricular Fibrillation patients when compared with 21/412 (five percent) of well-matched healthy controls (P-value=0.001). Furthermore, according to data from implanted cardioverter-defibrillators (ICD), 64 idiopathic Ventricular Fibrillation survivors with Early Repolarization had a greater rate of recurrence of Ventricular Fibrillation than 142 survivors of Ventricular Fibrillation in whom there was no Early Repolarization (41 % vs. 23 %, P=0.008) [3]. The ECGs of 45 patients with an unknown cause of Ventricular Fibrillation were compared to those of 124 gender and age-matched individuals in the control group and 121 athletes of young age by Rosso et al. They found that Early Repolarization was more prevalent in Ventricular Fibrillation patients than in the subjects of the control group (42 % versus 13 %, P=0.001). The finding was even more accurate for J-point elevation in the inferior ECG leads (27% vs. 8%, P=0.006) and the leads I-aVL (13% vs. 1%, P=0.009). Height of J-point in V4-6 occurred equally often in both matched controls and patients (7.3 % vs. 6.7 %, (P=0.86) [9]. In a study done in 2009, they found that ECGs at baseline in 11/19 (57.9%) patients with Ventricular Fibrillation showed Early Repolarization compared to 3.3% of controls, including 1,395 individuals [13].

Epidemiology

Early repolarization syndrome is usually seen in individuals who use cocaine, athletes, Hypertrophic obstructive Cardiomyopathy (HOCM), and patients with interventricular septal defect and/or hypertrophy [26-28]. Depending upon the criteria used for ECG diagnosis of ERP, the prevalence ranges between 3-24%. The majority is noted to be greater in younger individuals, people of African-American descent, athletic abilities, and patients prone to vagotonia [2,13].

Electrocardiogram of Early Repolarization

**Figure 1:**
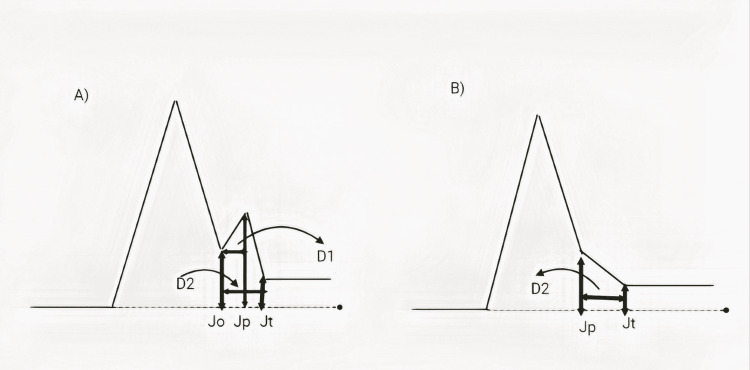
Early repolarization electrocardiogram terminology (A) J onset (Jo), J peak (Jp), and J termination (Jt), along with the durations D1 and D2, (B) Jp, Jt, and D2, are shown with a terminal QRS complex slur as described in the text of Macfarlane et al. [30].

According to Macfarlane et al., early repolarization occurs when all of the following criteria are met. 1) A prominent R-wave has a downslope end-QRS notch or slur. A notch, if present, should be above the baseline entirely, and Slur onset must also be above the baseline. 2) Jp is 0.1 mV in two or more consecutive 12-lead ECG leads, excluding V1-V3. 3)The duration of the QRS complex is 120 ms [29]. See Figure 1 attached.

In a patient resuscitated from an unexplained ventricular arrhythmia, the ECG indicative of ER is the QRS-ST junction upliftment above the baseline of more than one mm, which exhibits either a QRS slur or notch, ST-segment elevation with a concavity on the upper side, and T-waves which are prominent in two or more contiguous lateral and/or inferior electrocardiogram leads [2]. Antzelevitch et al. discovered three ER subtypes and a risk profile pattern: (1) The first type has Early Repolarisation in the V5 and V6 leads and has the lowest risk of malignant arrhythmias like VT/VF ; (2) The second type has Early Repolarisation in the inferior and inferolateral ECG leads and has more risk than type one for arrhythmias like VT/VF, and (3) The third type has Early Repolarisation in all ECG leads and has the highest risk of VT/VF. [30]. Another classification was provided by Juntilla et al. ER is defined as a J-point with an ST-segment elevation of more than one mm in two or more contiguous ECG leads. There are two varieties of J-point height above baseline ECG: 1) A Benign form is a J-point with a fast-sloping ST segment. 2). A malignant form is a J-point with a horizontal or falling ST segment [31]. The criteria for the diagnosis of early repolarization syndrome based on the HRS/EHRA/APHRS harmonious statement on the diagnosis and management of primary arrhythmia of hereditary origin along with ERS is summarised in Table 1 [32]. According to Sethi et al., malignant early repolarization is shown in an ECG with elevation at the J point, which is seen as slurring or notching in the inferior and/or lateral electrocardiogram leads and the uplifting ST-segment in most leads [33].

**Table 1:** ER expert consensus recommendations on early repolarization diagnosis ER: Early repolarization; ECG: Electrocardiogram; SCD: Sudden cardiac death HRS: Heart Rhythm Society, EHRA: European Heart Rhythm Association, APHRS: Asia Pacific Heart Rhythm Society [32].

HRS/EHRA/APHRS harmonious statement on the diagnosis and management of primary arrhythmia of hereditary origin along with ERS
1. Early repolarisation syndrome is defined as J-point elevation of more than or equal to one mm in more than or equal to two continuous inferior and/or lateral leads of a typical 12-lead electrocardiogram in a patient revived from an unknown cause of Ventricular Fibrillation/Polymorphic Ventricular Tachycardia.
2. Early repolarisation Syndrome can be diagnosed in a Sudden Cardiac Death victim with a negative post-mortem report and medical file review, as well as a prior ECG that shows J-point upliftment of more than or equal to one mm in more than or equal to two contiguous inferior and/or lateral leads of a standard 12-lead ECG.
3. An early repolarisation pattern can be identified when there is greater than or equal to one mm of the J-point upliftment in more than or equal to two contiguous inferior and/or lateral leads of a standard Electrocardiogram.

The mechanism behind Sudden Cardiac death in Early Repolarization Syndrome.

Experimental Studies

The precise mechanism of ER is uncertain. In 1991 it was postulated that transmural changes in the beginning stages of the action potential of cardiac muscle (phases 1 and 2) were the reason for the formation of the J wave on an ECG [34]. In 1996, they got compelling results in favor of this postulation in canine ventricular wedge preparations supplied by arterial blood [35]. The inference was a formation of a platform prone to the generation of arrhythmia by an unequal amplification of the current during repolarisation in the epicardial region of the myocardium due to a decrease in inward Ca+2 channel currents or Na+1 or an increase in outward K+ currents mediated by the Ito, IK-ATP, and IK-Ach channels. The dispersion of cardiac action potentials transmurally finally emerges as the onset and base for establishing phase 2 re-entry and Ventricular Tachycardia/Ventricular Fibrillation [2].

Genetics and ERS

As Early repolarization was falsely thought to be only a benign finding on ECG, genetic markers to differentiate between benign and arhythmic variants of ER are currently limited in research. The revelation of the relevance of genetic background in ERS was done by Haïssaguerre et al., revealing that 16 out of 100 individuals with Ventricular Fibrillation and Early Repolarisation have a family history of SCD. The population's high frequency of the genetic background underpinning the ER pattern is most likely influenced by multiple genes and environmental variables causing modification [3]. Using a candidate gene method, researchers discovered unusual monogenic variants of ER. Early Repolarisation on an ECG shows a change in the gradient of voltage transmurally between the epicardium and endocardium as a potential cause. This may occur by a rise in the IKACH, IKr, Ito, IKs, and IKATP currents or a fall in the ICaL or INa currents [36]. The first abnormality in a genetic variant was identified as an association with idiopathic Ventricular Fibrillation and inferolateral Early Repolarisation in a 14-year-old girl with Ventricular Fibrillation with Early Repolarization syndrome. It was found in a subunit Kir6.1 of the IKATP channel, whose function is the formation of pores, utilizing a unique gene method [37]. Examination of 101 individuals with J-wave syndromes, including 87 patients with Brugada syndrome and 14 patients with Early Repolarization syndrome, was done in 2010. One case in each group had the same missense variant, KCNJ8- S422L.47. This was supported by the discovery of KCNJ8, a new gene that increases susceptibility to J-wave syndromes [38]. A missense mutation in the two subunits of the calcium channel of the L-type in the cardiac tissue in Early Repolarisation syndrome patients was found in 2010. There are presently no expression studies for this variant available [39].

Depolarization or Early Repolarization?

The "J-wave syndromes" (Brugada and inferolateral J waves) are the subject of continuous debate as to whether they are caused by repolarization or depolarization anomalies. Although early repolarization nomenclature appears appropriate in situations of early repolarisations, it is inaccurate when depolarization that happens late is the cause for a J-wave. As a result, the name inferolateral J wave may be more appropriate and general [40]. Repolarization anomalies have long been recognized as the primary substrate in inferolateral J waves. On the other hand, termination in a slurred fashion of the QRS complex is a hallmark of late activation in SHD (known as epsilon wave or peri-infarction block). Analogous to the delta wave in Wolff-Parkinson-White syndrome, which shows "pre-excited" myocardial, the J wave here suggests "post excited" myocardium [41]. According to a study conducted by Nademanee et al., the J wave on an ECG is a symbol of either delayed depolarization or repolarization that is early in the inferior part of the ventricle. In patients with concurrent Brugada Syndrome, delayed depolarization of the inferior part of the myocardium reliably generated inferior and lateral J waves (spontaneous or induced by a Na+ channel blocker). Delay in depolarization was the reason for inferolateral J waves in 24% of cases, whereas Early Repolarisation was the reason in 76% of cases in patients that didn't have BrS [42]. In research conducted by Haissaguerre M, Ventricular Fibrillation drivers were mapped using a noninvasive approach, revealing prominent origins in the inferior region of the cardiac muscle during the early phases of Ventricular Fibrillation. Ventricular Fibrillation is predominantly related to origins in the inferior and anterior right ventricles in subjects with J waves indicating delayed depolarization. VF, on the other hand, was linked to drivers in the inferior septum and surrounding areas during early repolarization. It is worth noting that the epicardial area that lies over the inferior part of the septum might be the point when activity from the fascicle on the posterior part of the Purkinje system makes a breakthrough [43]. The therapeutic potential of identifying J-wave subtypes is an instant gain. As with BrS and other SHDs, substrate ablation targeting the delayed electrograms is possible in patients with late depolarization J waves. Although it is unknown whether ablation of aberrant repolarizing tissue is appropriate and safe in individuals with ER J waves, trigger ablation is a viable alternative when antiarrhythmic medications, notable quinidine, have failed [40].

Risk Stratification

Patients with ERS can be split into two groups based on their clinical presentation. Those who present with symptoms such as syncope and cardiac arrest survivors fall into the first category. According to Abe et al., the Early Repolarisation pattern was noted in 18.5 percent of syncope patients compared to two percent of healthy controls, implying an almost 10-fold increase in the risk of syncope in individuals with Early Repolarization Syndrome [44]. Recurrent cardiac episodes are particularly likely in this population, although the risk is slightly increased compared to the general population. The second and most prevalent type consists of asymptomatic individuals with an Early Repolarisation change on their ECG by chance. This cohort of people with incidental ER patterns is less likely to experience negative cardiac symptoms. The problem lies in differentiating individuals likely to have SCD from those who will run a safe course of ERS [36].

Risk in Asymptomatic Subjects

J wave incidence in people with athletic abilities has been estimated to be present in 44 out of every 100 athletes. No arrhythmic occurrences were seen in a sample of 704 people with athletic abilities (J wave present in 14%) during a six-year course of research [29]. When followed up for an extended period, adults with J-waves of a remarkably young age revealed no link to an increased mortality risk [45]. J-point elevation was discovered to be associated with interventricular-septal hypertrophy and a potential connection with exercise-induced hypertrophy [45,46]. J waves have also been linked to false tendons, possibly due to abnormal depolarization in the Purkinje system due to localized strain [47]. Although J waves are widespread in African Americans, no additional risk exists in this subgroup, but a more significant risk is thought to exist in Asian groups [11,48]. It is worth noting that the definition of Early Repolarisation differs in different literature reviews and original articles regarding the pattern of ST-elevation and the amplitude of the J-wave used for the inclusion, which influences the prevalence and the declared prognostic value [40].

Risk in Association with Structural Heart Disease (SHD)

According to several investigations, J waves associated with SHD have been linked to an increased risk of ventricular arrhythmias but not non-arrhythmic cardiac events [49-51]. Inferior leads with J-waves, a notched J-wave, and a falling or horizontal ST-segment were associated with a greater risk in 19 studies involving 7268 SHD patients [52]. In individuals with chronic CAD or dilated/hypertrophic cardiomyopathy, inferior lead J-waves were also linked to an elevated risk [51-53]. Thus, inferior J waves in SHD patients raise the risk of arrhythmic events. J waves also increase arrhythmias and their association with co-existing electrocardiogram abnormalities (e.g., Brugada, prolonged QT, and short QT syndromes) [54].

J waves and Idiopathic VF

Many studies have found an increased incidence of J-wave patterns in people with idiopathic VF. In recent Korean research, J waves were seen in 35/81 (43%) subjects with idiopathic Ventricular Fibrillation and were linked to a greater likelihood of recurrences [55]. It is estimated that 3:100,000 people have inferolateral J waves with idiopathic VF [3,9-11, 56]. Compared with the population's prevalence of one percent to 24 percent, this reflects a minimal absolute risk. Hence, most people with ER have a low risk of arrhythmic events; as a result, asymptomatic patients with no family history of SCD should rest easy [57,58].

Several ECG indicators have been studied in connection to prognosis in symptomatic individuals. On the other hand, the J-ST pattern's spontaneous fluctuation is an issue with limitations. Clinical attributes such as sex and ethnic background have varied prognostic importance results. It is a well-known fact that history in the family of SCD has been related to an elevated risk of SCD and is likely to increase the individual's risk [59]. The predictive relevance of QRS slurring versus notching, which frequently co-exists or changes over time, is unknown. The following factors have been linked to a higher chance of developing malignant arrhythmias: (1) ST-segment that is horizontal or descending in the inferior leads, rather than an ST segment going upwards [10,11,45,56]; nonetheless, the occurrence in controls of this ST pattern might be at 3%, lowering its specificity; (2) a larger J wave amplitude (2.0 mm) in the inferior leads [3,9]; a broad ECG pattern including both anterior and inferior leads [3,60].

Amplification of inferolateral J waves is a constant finding during malignant arrhythmias, which fades once the arrhythmia is terminated spontaneously or pharmacologically [3,22]. A larger J-wave amplitude obtained immediately after a syncope (compared to earlier or later ECGs) may indicate the presence of a malignant arrhythmia. We believe that the J wave variability with longer cycle durations (post pause) is an essential indicator of electrical susceptibility. No specialized methods exist for testing the dynamicity of J-wave patterns in susceptible individuals (for example, sodium blocker provocation in Brugada Syndrome). A Valsalva maneuver or Holter monitoring might be helpful when determining the J-wave dynamics on a 12-lead ECG during cycle length fluctuations. Finally, during electrophysiological research, VF induction procedures are ineffective. VF inducibility could not predict the occurrence of future arrhythmias in a multicenter trial of 81 patients who were monitored with implanted cardioverter-defibrillator interrogations [61].

In conclusion, no risk stratification that is strong clinically can be undertaken to find the tiny group of patients at high risk and enable primary prevention, based on many published reviews. Genetic variant analysis or pharmacologic testing may become more critical for prognosis [62]. Currently, the implant choice of an implanted cardioverter-defibrillator (ICD) in high-risk patients or a loop recorder in medium-risk patients is based on the seriousness of clinical factors and ECG patterns: T-wave inversion, the height of J-wave, J-wave pattern spatial extent, and dynamic variations in the J wave pattern [29,57,58]. The risk factors stratification is based on clinical features, and ECG findings are summarised in tables 2 and 3.

**Table 2:** Stratified view of risk factors based on clinical features in patients with ERS SCD: Sudden Cardiac Death [40]

CLINICAL FEATURES OF ERS WITH ASCENDING RISK FOR ARRHYTHMIA
1. Family History of SCD.
2. Syncope with severe criteria: Agonal breathing, apnea, convulsions, injury, urine loss.
3. Cardiac Arrest

**Table 3:** Stratified risk factors based on ECG findings in patients with ERS LQT = long QT; SHD = structural heart disease; VPB = ventricular premature beat [40].

ECG Findings associated with ascending risk for arrhythmia in ERS
1. Horizontal/descending ST Segment
2. The Amplitude of greater than two mV of the J point
3. Associated pathology: Brugada Syndrome, SHD, Fragmented QRS- LQT
4. Widespread J wave
5. Dynamic J wave changes
6. Short coupled VPBs

TREATMENT

The early repolarization pattern is a harmless bystander finding in most individuals and has no associated specific indications or symptoms. There is currently no risk classification technique for asymptomatic people with Early Repolarization patterns in the normal population or within blood relatives with Early Repolarization patterns that would allow for the recognition of Early Repolarization patterns in individuals who could be candidates for therapy. According to the current agreement, these individuals do not require additional testing or treatment [18]. SCD survivors due to idiopathic VF reported the risk of recurrent VF was between 22 percent and 37 percent at two to four years of follow-up [63]. These individuals with idiopathic VF have a favorable prognosis for long-term survival if VF is treated since they have no cardiac structural problems. As a result, an implanted cardioverter-defibrillator (ICD) is the best treatment option for such individuals [63-65].

Patients with Ventricular Fibrillation and Early Repolarisation syndrome during a five-year follow-up study had a greater frequency of Ventricular Fibrillation recurrence than Ventricular Fibrillation patients without Early Repolarisation (43 % vs. 23 %, P<0.001) [3]. Isoproterenol was used with good results for the acute suppression of Ventricular Fibrillation in a 122-patient cohort study (90 male adults, with a mean age of 37.12 years) with Early Repolarisation in the inferior and lateral ECG leads and with more than three episodes of unexplainable Ventricular Fibrillation (including patients with the electrical storm) [66]. In terms of prolonged treatment, quinidine has successfully decreased VF recurrence [66]. Gurabi et al. recently published encouraging findings showing that milrinone and cilostazol are similar to quinidine in reducing hypothermia-induced VT/VF in an experimental model of the left ventricle in a canine [67]. People with syncope, Malignant Early repolarisation patterns, and a strong family history of SCD fall somewhere in a grey area where there are no definite standards. According to current recommendations, an Implantable Cardioverter Defibrillator(ICD) may be helpful in people in whom the risk of idiopathic syncope is high [36]. Table 4 summarises the HRS/EHRA/APHRS harmonious statement on the identification and handling of inherited primary arrhythmia syndromes and suggestions for interventions in Early Repolarization Syndrome [32].

**Table 4:** Expert consensus recommendations on early repolarization therapeutic interventions Recommendations for therapeutic therapies in early repolarization syndrome from the HRS/EHRA/APHRS consensus statement on the diagnosis and management of primary hereditary arrhythmia syndromes [32]. HRS: Heart Rhythm Society, EHRA: European Heart Rhythm Association, APHRS: Asia Pacific Heart Rhythm Society

Expert consensus recommendations on early repolarization therapeutic interventions	
Class I	1. Implantable Cardioverter Defibrillator implantation is advised in individuals with Early Repolarisation syndrome who have survived an arrest of the heart.
Class IIa	2. Isoproterenol infusion may be beneficial in reducing arrhythmias in individuals with Early Repolarisation syndrome.
	3. Quinidine, in addition to an Implantable Cardioverter Defibrillator, may be beneficial for secondary Ventricular Fibrillation prophylaxis in individuals with Early Repolarization syndrome.
Class IIb	4. In symptomatic family members of Early Repolarization syndrome patients with a history of syncope and ST-segment elevation of greater than one mm in two or more inferior or lateral leads, Implantable Cardioverter Defibrillator implantation may be explored.
	5. In asymptomatic people with a high-risk Early Repolarization ECG pattern (high J-wave amplitude, horizontal/descending ST-segment) and a significant family history of young age unexplained sudden death with or without a genetic mutation, Implantable Cardioverter Defibrillator implantation may be explored.
Class III	6. Implantation of an Implantable Cardioverter Defibrillator is not advised in asymptomatic individuals with an isolated Early Repolarization ECG pattern.

## Conclusions

Early repolarization, previously thought to be a standard variant, is now a significant risk factor for Sudden Cardiac death, especially in patients with inferolateral J-waves and Structural Heart diseases. The increased risk of sudden cardiac death is due to an unequal elaboration of the repolarisation electrical wave in the epicardial myocardium due to a decrease in inward Na+ or Ca+2 channel currents or an increase in outward K+ currents mediated by the IK-ATP, Ito, and IK-Ach channels. The dispersion of cardiac action potentials transmurally eventually emerges as the onset and base for the formation of phase 2 re-entry and thus precipitates into Ventricular Tachycardia /Ventricular Fibrillation. Patients with Early Repolarization who have idiopathic syncope, a history in the family of SCD, or unexplained ventricular arrhythmias should be evaluated carefully. A J wave upliftment above the baseline of > 0.2mV, a J-wave present in all ECG leads, and a notched J-wave in the inferolateral leads should be investigated prospectively for their prognostic relevance. There is currently no risk classification technique for asymptomatic people with early repolarization patterns in the general population or within blood relatives with Early Repolarization patterns that would allow for identifying individuals with increased risk of Early Repolarization patterns who could be candidates for therapy. According to the current agreement, these individuals do not require any additional testing or treatment because a considerable number of people meet these characteristics but do not appear to be at an increased risk of arrhythmias; future research is required to determine how to point out the asymptomatic individuals who are at an increased risk and what measures may be taken to avoid it. 
